# The Influence of the Social Context on Motivation towards the Practice of Physical Activity and the Intention to be Physically Active

**DOI:** 10.3390/ijerph16214212

**Published:** 2019-10-30

**Authors:** Rubén Trigueros, José M. Aguilar-Parra, Adolfo J. Cangas, José María Fernández-Batanero, Joaquín F. Álvarez

**Affiliations:** 1 Department of Psychology, Hum-878 Research Team, Health Research Centre, University of Almería, 04120 Almería, Spain; jalvarez@ual.es; 2 Department of Psychology, Hum-760 Research Team, Health Research Centre, University of Almería, 04120 Almería, Spain; ajcangas@ual.es; 3 Department of Education, University of Seville, 41011 Seville, Spain; batanero@us.es

**Keywords:** adolescence, physical activity, social context, motivation, school

## Abstract

According to WHO data, only around 20% of adolescents participate in physical activity (PA) during their free time. The social context can act as a support for adolescents to do PA, given the effect that both parents, friends and teachers have on young people’s behaviour owing to the large amount of time and influence they have on them. Therefore, the aim of this study is to analyse the role of the social context on adolescents’ motivation to practise PA and their intention to be physically active. This study involved the participation of students in compulsory and post-compulsory secondary education. Several statistical analyses were performed, including three confirmatory factorial analyses of the scales and a structural equations model that explains the causal relationships between the variables. The results showed how support for autonomy in the social context positively predicts autonomous motivation, whereas the psychological control of the social context negatively predicts it. Autonomous motivation positively predicted intent, attitude, behavioural control and subjective norms, and consequently, the practice of physical activity. In short, the study showed how the three validated scales have adequate goodness-of-fit indices while the structural equations model demonstrated the influence of the social context on the student’s motivational processes and the adoption of active life habits.

## 1. Introduction

The benefits associated with the practice of regular physical activity (PA) at the psychological, physical and emotional levels have been widely demonstrated [1], with a sedentary lifestyle being an important risk factor in the development of chronic diseases, obesity, depression and anxiety [2]. However, most of the adult population is sedentary or not sufficiently active [1]. According to WHO [3], 80% of adolescents do not engage in any type of physical/sports activity in their spare time. Their data has also shown [1] that the prevalence of obesity in our country has increased from 3% to 12% in boys and from 2% to 8% in girls; it is between the ages of 15 and 18 when one finds the highest drop-off rate in sports activity as adolescents’ interests shift towards other leisure practices as they grow older [4]. That is why the social context can act as a support for adolescents to do PA, given the effect that both parents, friends and teachers have on young people’s behaviour, in large part because of the time spent with them and the influence they have on them [5]. Therefore, the objective of this study is to analyse the role of social context in motivating young people to engage in PA and their intention to be physically active.

In recent years, various studies have emerged that have focused on determining the psychosocial variables involved in physical practice and performance, as well as the influence of each of them [6,7]. Studying the contextual variables involved in sports is very useful in influencing and improving one’s living conditions (for health, affiliation, and fun, etc.). Among these variables, the one that stands out is the influence of people in the immediate environment, i.e., parents, peers and teachers [8,9]. The family is the first affective, socializing and educational agent of direct influence enabling the individual’s personal development and their adaptation to society as a whole. In this sense, parents constitute the nucleus from which the first experiences of sports socialization originate, their attitudes being determinant with regard to the practice of physical activity [10]. Parents have also been found to have a major influence on their children’s motivation towards certain activities, such as school performance, offering support and generating stimulating environments [11]. Likewise, friendships play an important role in all respects, including in the encouraging or censoring of PA practice. Friends enable personal growth, the development of self-esteem, a sense of personal worth, the building of one’s own identity and they even help aspects beneficial to health through psychophysiological processes [12]. It is also through friendships that the child and adolescent acquires a sense of integration and security [13,14]. Finally, the physical education (PE) teacher plays a vital role, being necessary to ensure a sense of competence in the students, while encouraging motivation, challenging them to invest effort and energy in decision-making, adapting content and activities to the students’ needs and rewarding them for their achievements, amongst other things [15]. Teachers, together with teammates, play a prominent role when children start sport and PA [16], with the teacher’s influence even going beyond the sporting realm [17].

Self-Determination Theory (SDT) describes how human behaviour is influenced by the social and interpersonal environment, suggesting that the role adopted by social agents (parents, friends and teachers) might come from two sides, supporting autonomy versus a controlling style [18]. In this regard, support for autonomy is a key feature in the interpersonal relationship, which encourages the adolescent’s internal motivation towards certain behaviours because, through the promotion of personal initiative, offering relevant objectives and the use of minimal contingencies will promote the adolescent’s sense of support [19]. In contrast, the use of a controlling style by social agents, which involves acting in an authoritarian and coercive manner, using unattainable objectives and not enhancing personal initiative, will mean decreased motivation and enjoyment for the adolescent and in the internal locus of those behaviours that it manifests [20]. However, most of the studies existing to date have focused solely on the role of the teacher in relation to their students’ motivations towards Physical Education classes and towards the practice of PA outside the school, without considering the relevant role of parents and friends. Various studies focusing on the teacher’s role have shown the existence of a positive relationship between the support for autonomy and self-determined motivation, as well as towards adolescents engaging in physical activity and sport outside of school hours [18,21,22]. Conversely, other studies have shown how the use of an interpersonal controlling style by the teacher (as perceived by PE students) affects the motivation of the adolescents since the teaching style was found to be linked to external motivation [23,24].

Traditionally, the Perceived Autonomy Support Scale for Exercise Settings (PASSES; [25]), validated and adapted to the Spanish context by Moreno, Parra and González-Cutre [26] has been used to measure support for autonomy as perceived by adolescents, while the Psychologically Controlling Teaching Scale (PCT) of Soenens, Sierens, Vansteenkiste, Dochy and Goossens [27] validated and adapted to the Spanish context recently by Trigueros, Aguilar-Parra, González and Cangas [24] has been employed to measure perceived psychological control. These scales are a series of effective tools with which to measure the teacher’s teaching style; therefore, it is necessary to adapt and validate both scales to the interpersonal style of parents and friends with respect to adolescents, given the influence they exert on the intentions and behaviours they manifest. In addition, we propose to combine the two scales into a single tool that allows us to evaluate the dichotomy between the two styles—support for autonomy versus psychological control—since each scale only evaluates a single aspect. In this way, the duality in similar questionnaires in the SDT is maintained (see, [28,29]).

In this way, depending on if the influence of the context is controlling or autonomy support, will have an effect on the adolescent’s motivation towards the practice of PA. Accordingly, motivation is understood as a directional process, “something that drives action” [30], which may be linked to an internal or external component [30]. Therefore, motivation can be intrinsic or external. The first could be defined as the tendency to develop one’s own skills, to face challenges and engage in new activities voluntarily, bypassing environmental reinforcements [31], aspects that are linked to supporting autonomy. Intrinsically motivated adolescents engage in various activities out of interest and the curiosity to develop them. Conversely, the source of extrinsic motivation is external to the person, dependent on certain environmental conditions being met, such as obtaining some kind of benefit or avoiding adverse consequences, which is linked to the control of behaviour [32].

Motivation is the impulse towards a certain action, whereas decision-making is something more complex. One of the explanatory models most widely used in recent years because of its predictive character is the Theory of Planned Behaviour (TPB; [33,34]), which integrates the various psychological variables by which people make systematic use of information and consider the consequences of their actions before carrying them out. The immediate determinant of exercise behaviour is the intention to perform it, which itself is determined by three factors: (a) the personal attitude towards the behaviour, the confluence of the person’s beliefs regarding the results of the intended behaviour and the assessment of such results and experience; (b) the subjective social norm, i.e., the normative beliefs as to whether or not significant people consider that the adolescent should carry out the behaviour, and the motivation to please such pressures, each of these factors having a relative weight that needs to be specified; and finally, (c) the perception of behavioural control that the person has in making decisions and acting accordingly in a given situation.

Based on the model, for the adolescent’s physical exercise behaviour to occur successfully, the person must have a positive attitude towards the sport, considering the benefits for their health, positively valuing the state of well-being that is generated (beliefs about results, assessment and experience) and consider that their influence group (parents, teachers, friends) positively values this type of practice or even displays rejection attitudes for not practicing sport, that the opinion of others is very valuable, (beliefs about others, the motivation to fulfil), as well as being considered sufficiently capable of carrying out the sporting activity (the perception of control).

TPB and SDT have been used by researchers to try to explain the processes that underlie people’s motivated behaviour [35]. In this sense, the TPB offers an approach to understanding people’s belief system, in which a certain behaviour will lead to certain results. Instead, SDT offers explanations of the origin of behaviour based on social cognitive theories [30]. Thus, through the integration of both theories, beliefs about outcomes could be interpreted as autonomous or controlled reasons for engaging in health-related behaviors (i.e., “I do exercise for a better quality of life”). On this basis, SDT suggests that motivation to engage in health-related behaviors for autonomous or control reasons predisposes individuals to form beliefs congruent with these motives. Therefore, self-determined motives are a predictor of attitudes, subjective norms and perceived behaviour control. Attitudes and perceived behaviour control are, in turn, proximal predictors of the formation of intentions to adopt future health-related behaviours [36].

For this reason, we present two studies: (1) to adapt and validate the Perceived Support Scale in Exercise Contexts for parents, friends and teachers in order to measure the influence of the social context on adolescent motivation (see, Appendix A); (2) to analyse the influence of this social context on motivation towards PA, the intention to be physically active through TPB and the amount of weekly PA—both using a structural equations model (SEM). In this way, we will attempt to explore other adolescent weighting variables included in the TPB framework to develop a model for making predictions regarding PA practice.

## 2. Study Method 1

### 2.1. Participants

To be able to validate and adapt the scales, 428 secondary school students (211 boys and 217 girls) were asked to participate, aged between 13 and 19 years (M = 15.30; SD = 1.15), all from Almeria province.

The sampling used was accidental non-probabilistic (convenience sampling) in terms of those schools and students that were accessed.

### 2.2. Instruments

The Scale of Perceived Support in Exercise Contexts on the part of the teacher (SPSEC-T)—this scale comprises 19 items spread over two factors: 12 items for the support of autonomy and 7 items for psychological control. This scale is preceded by the following statement: ‘In relation to the practice of physical activity...’ The scale was responded to using a Likert scale from 1 (totally disagree) to 7 (totally agree).

The Scale of Perceived Support in the Exercise Context on the part of Parents (SPSEC-P)—this scale comprises 19 items spread over two factors: 12 items for the support of autonomy and 7 items for psychological control. This scale is preceded by the following statement: ‘In relation to the practice of physical activity...’ The scale was responded to using a Likert scale from 1 (totally disagree) to 7 (totally agree).

The Scale of Perceived Support in the Exercise Context on the part of Friends (SPSEC-F)—this scale comprises 19 items spread over two factors: 12 items for the support of autonomy and 7 items for psychological control. This scale is preceded by the following statement: ‘In relation to the practice of physical activity...’ The scale was responded to using a Likert scale from 1 (totally disagree) to 7 (totally agree).

### 2.3. Procedure

Initially, the scale measuring the support given by the teacher for student autonomy in exercise contexts and that concerning the psychological control exerted by the teacher as perceived by the students were included in the same questionnaire. Both questionnaires were validated in the Spanish context. The next step was to take out the references made to teachers and replace them with references to the family (the first questionnaire) and to friends (the second questionnaire). Thus, with the two scales integrated, we proceeded to validate the scales of perceived support by parents and friends in the context of exercise.

Once the questionnaires were prepared, the heads of the participating schools were contacted, and through them the physical education teachers, who were also informed of the purpose of the research and asked to collaborate in having informed consent granted. Underage students were required to have the parents authorize their participation. Before administering the scale to all the participants, it was completed by a small group of students to ensure that all the items were correctly understood. The questionnaire was administered under the supervision of an expert survey taker, a member of the research group, who explained the procedure and resolved any doubts that arose while the students completed it. The estimated time for completing the questionnaire was around 15 minu. The survey was carried out in the second week of January in the academic year 2018/2019, in the different secondary classes.

### 2.4. Data Analysis

In order to determine the validity and reliability of the SPSEC-T, SPSEC-P and SPSEC-F in the Spanish context, the psychometric properties of the questionnaires were analysed. First, a confirmatory factor analysis (CFA) was performed to test the factorial structures. Secondly, multigroup analyses were carried out to analyse age and gender invariance in order to determine whether the questionnaire was equally understood by the boys and girls. Subsequently, a descriptive statistics analysis, an analysis of internal consistency (Cronbach’s alpha) was performed to test the instrument’s reliability. The SPSS 23.0 (IBM, Armonk, NY, USA) and AMOS 19.0 (IBM, Armonk, NY, USA) statistical packages were used for data analysis.

For the CFA, the maximum likelihood estimation method was used in conjunction with the bootstrapping procedure. To do this, a set of goodness-of-fit indices was considered to either accept or reject the tested model: *χ*^2^/*df*, CFI (*Comparative Fit Index*), IFI (*Incremental Fit Index*), RMSEA (*Root Mean Square Error of Approximation*) plus its 90% confidence interval (CI), and SRMR (*Standardized Root Mean Square Residual*). Since the *χ*^2^ is very sensitive to sample size [37], the *χ*^2^/*df* was used, with values less than 5 deemed acceptable [38]. The incremental indices (CFI and IFI) showed a good fit, with values equal to or greater than 0.90 [39], while error indices (RMSEA and SRMR) were considered acceptable, with values equal to or less than 0.080 [40,41].

## 3. Study 1 Results

### 3.1. Descriptive Statistics, Reliability Analysis and Bivariate Correlations

The descriptive statistics, bivariate correlations and reliability analysis by Cronbach’s α can be seen in Table 1. The internal consistency analysis revealed Cronbach’s α values greater than 0.80 for each of the variables.

### 3.2. Confirmatory Factor Analysis

**Figure 1:**
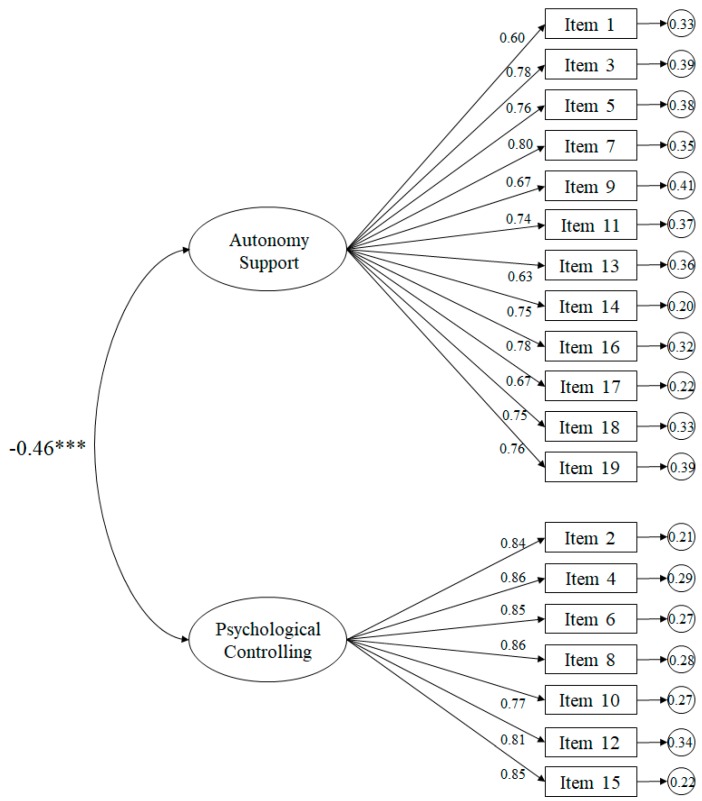
Confirmatory factor analysis of the SPSEC-T. The ellipses represent the factors and the rectangles represent the various items. Residual variances are shown in the small circles.

The fit indices for the tested model (Figure 1) related to the teachers revealed appropriate fits: χ^2^ (151. *N* = 428) = 560.82, *p* < 0.001; χ^2^/*df* = 3.71; CFI = 0.94; IFI = 0.94; RMSEA = 0.080 (IC 90% = 0.073–0.087); SRMR = 0.041. The standardized regression weights ranged from 0.60 to 0.86, and were statistically significant (*p* < 0.001). The correlation between the support for autonomy and psychological control factors was −0.46 and was likewise statistically significant (*p* < 0.001).

**Figure 2:**
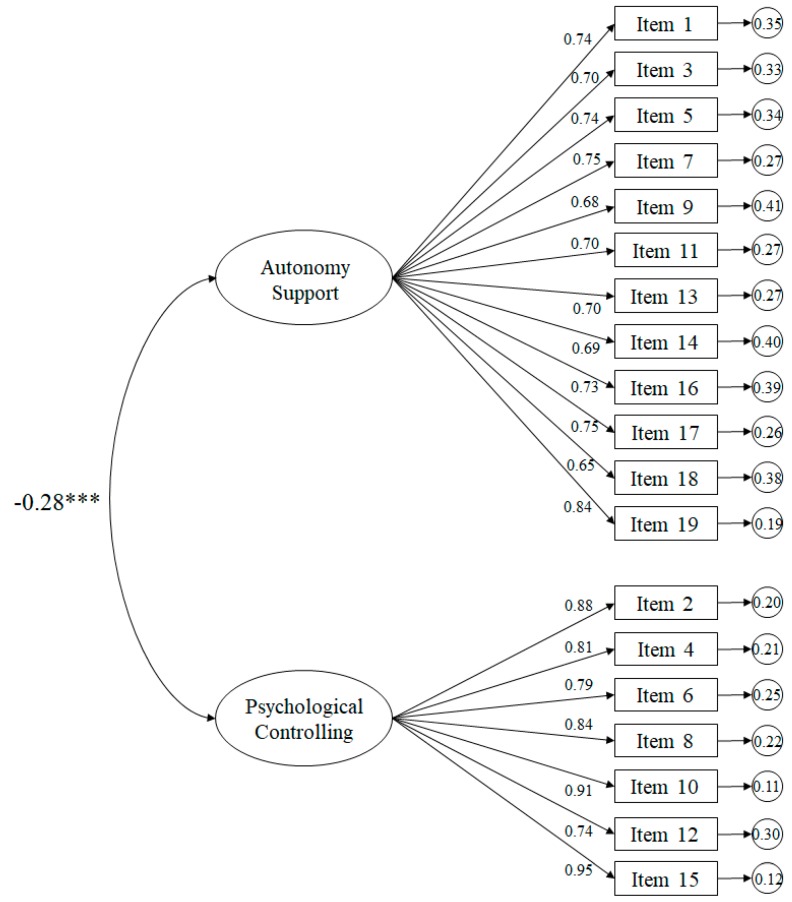
Confirmatory factor analysis of the SPSEC-P. The ellipses represent the factors and the rectangles represent the various items. Residual variances are shown in the small circles.

The fit indices of the tested model (Figure 2) related to the parents revealed appropriate fits: χ^2^ (151. *N* = 428) = 555.34, *p* < 0.001; χ^2^/*df* = 3.68; CFI = 0.93; IFI = 0.93; RMSEA = 0.079 (IC 90% = 0.072–0.086); SRMR = 0.033. The standardized regression weights ranged from 0.65 to 0.95, and were statistically significant (*p* < 0.001). The correlation between the support for autonomy and psychological control factors was −0.28 and was likewise statistically significant (*p* < 0.001).

**Figure 3:**
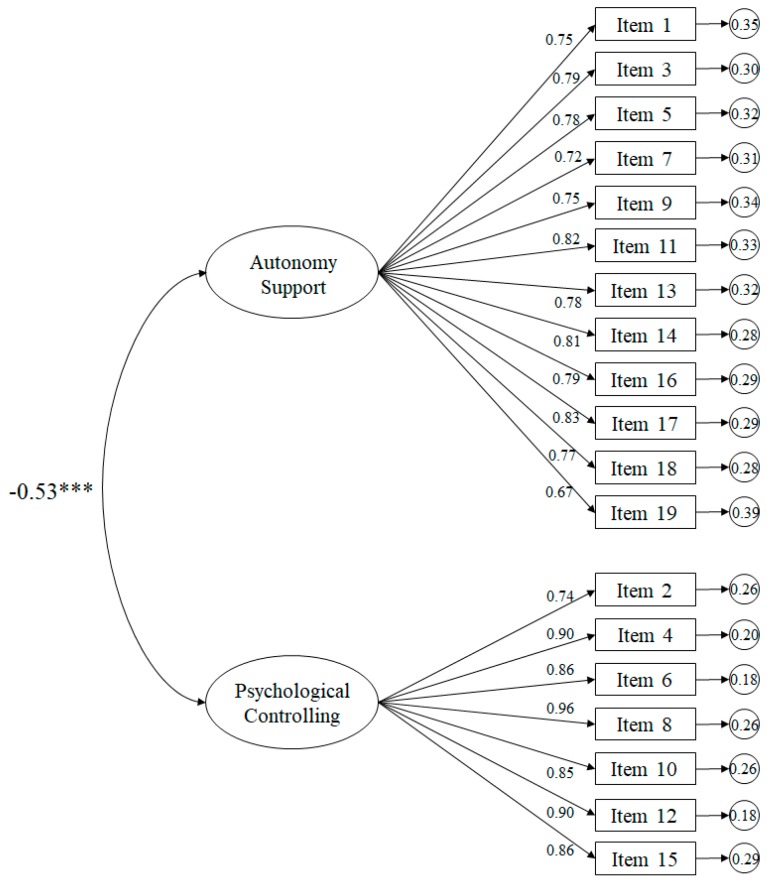
Confirmatory factor analysis of the SPSEC-F. The ellipses represent the factors and the rectangles represent the various items. Residual variances are shown in the small circles.

The fit indices of the tested model related to friends (Figure 3) revealed appropriate fits: χ^2^ (151. *N* = 428) = 552.45, *p* < 0.001; χ^2^/*df* = 3.66; CFI = 0.96; IFI = 0.96; RMSEA = 0.074 (IC 90% = 0.073–0.084); SRMR = 0.040. The standardized regression weights ranged from 0.67 to 0.96 and were statistically significant (*p* < 0.001). The correlation between the support for autonomy and the psychological control factors was −0.53 and was likewise statistically significant (*p* < 0.001).

### 3.3. Gender Invariance Analysis

As shown in Table 2 and Table 3, a multigroup analysis was performed for each of the subscales to understand whether the factorial structure of both models demonstrated invariance across gender and age. As shown in Table 2 and Table 3, in the parental support section, no significant statistical differences were observed in the *χ*^2^ statistic between Model 1 (the unrestricted model), Model 2 (the measurement weight invariance model) and Model 3 (the invariant structural covariance model). The results did show significant differences between Model 1 and Model 4 (the residual invariance measurement model). Conversely, also in Table 2 and Table 3, in the support on the part of friends section, no significant differences were observed in the *χ*^2^ statistic between Model 1 and Model 2. The results did show significant differences between Model 1 and Models 3 and 4. The absence of significant differences between Model 1 and Model 2 supposes a minimum criterion for accepting that the model structure is invariant across gender and age [42].

## 4. Study Method 2

### 4.1. Participants

In Study 2, 653 high school students (311 boys and 342 girls) participated, aged between 13 and 19 years (M = 15.56; DT = 1.23), coming from the province of Almería. The sampling used was accidental non-probabilistic (convenience sampling) in terms of those schools and students that were accessed.

### 4.2. Instruments

The Scale of Perceived Support in Exercise Contexts on the part of the teacher (SPSEC-T)—this scale comprises 19 items spread over two factors: 12 items for the support of autonomy and 7 items for psychological control. This scale is preceded by the following statement: ‘In relation to the practice of physical activity...’ The scale was responded to using a Likert scale from 1 (totally disagree) to 7 (totally agree). 

The Scale of Perceived Support in the Exercise Context on the part of Parents (SPSEC-P)—this scale comprises 19 items spread over two factors: 12 items for the support of autonomy and 7 items for psychological control. This scale is preceded by the following statement: ‘In relation to the practice of physical activity...’ The scale was responded to using a Likert scale from 1 (totally disagree) to 7 (totally agree).

The Scale of Perceived Support in the Exercise Context on the part of Friends (SPSEC-F)—this scale comprises 19 items spread over two factors: 12 items for the support of autonomy and 7 items for psychological control. This scale is preceded by the following statement: ‘In relation to the practice of physical activity...’ The scale was responded to using a Likert scale from 1 (totally disagree) to 7 (totally agree).

Planned behavior—The Theory of Planned Behaviour Questionnaire by González, López, Marcos, and Rodríguez-Marín [43] was used; this comprises 20 items divided between four factors: subjective norm (4 items), intention (4 items), perceived behavioural control (5 items) and attitude (7 items). The questionnaire leads with the following statement ‘For me, to exercise at least 6 times in the next two weeks would be...’. Each item was answered with a Likert scale ranging from 1 (totally disagree) to 7 (totally agree), except for one subjective norm factor item ranging from 1 (no control) to 7 (a lot of control).

Motivation towards PA—The Behavioural Regulation in Exercise Questionnaire (BREQ-3) by Wilson, Rodgers, Loitz, and Scime [44] validated and adapted to the Spanish context by González-Cutre, Sicilia, and Fernández [45] was used to measure the motivation of exercise practitioners. This questionnaire consists of 23 items related to intrinsic regulation spread across six factors: intrinsic motivation, integrated regulation, identified regulation, introjected regulation, external regulation and demotivation. Students responded using a Likert scale ranging from 0 (not true at all) to 4 (totally true), which was headed by the statement ‘I practice physical exercise...’.

To evaluated autonomous motivation, the Self-Determination Index [46] was employed, calculated from the following formula: 3 x intrinsic motivation, 2 x integrated regulation, 1 x identified regulation, −1x introjected regulation, −2 x external regulation and −3 x amotivation. This index has proven valid and reliable in several works. It is used to obtain a value for quantifying the level of self-determination.

PA practice frequency—to measure the amount of physical activity practice, high school students were asked for the days of the week that they normally practiced physical activity.

### 4.3. Procedure

The people in charge at the participating schools were contacted, they were informed about the purpose of the research and their collaboration was requested. Authorization was required from the parents or legal guardians of underage students in order for them to participate in the study. The questionnaire was administered under the supervision of an expert survey taker, a member of the research group, who explained the procedure and resolved any doubts that arose while the students completed it. The estimated time for completing the questionnaire was around 15 min. The survey was carried out in the third week of February in the academic year 2018/2019, in the different secondary classes.

This study was carried out in accordance with the recommendations of the American Psychology Association. The entire experiment was conducted in accordance with the Helsinki Declaration. Ethical approval was obtained from the Research Ethics Committee of the University of Almeria, Spain (Ref. UALBIO 2019/014).

### 4.4. Data Analysis

Descriptive statistical analyses, bivariate correlations and reliability analyses were performed using the SPSS v24 Statistical Programme. In addition, a structural equation model (SEM) (AMOS v19) was constructed.

**Figure 4:**
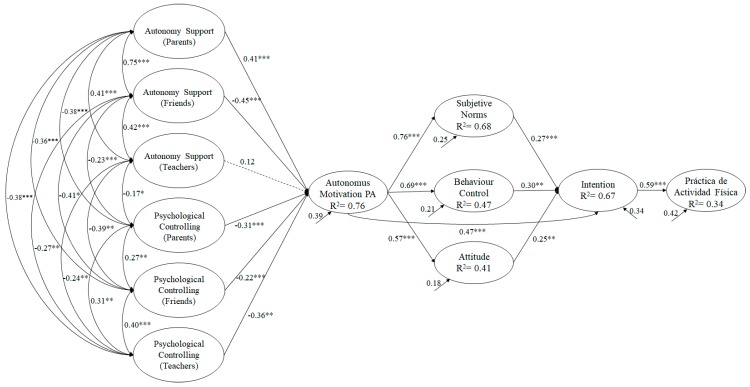
A hypothesized model showing the importance of social context on motivation and planned behaviour.

To analyse the hypothesized model (Figure 4), the maximum likelihood estimation method was used in conjunction with the bootstrapping procedure. To do this, a set of fit indices was taken into consideration so as to accept or reject the tested model: *χ*^2^/*df*, CFI (Comparative Fit Index), IFI (Incremental Fit Index), RMSEA (Root Mean Square Error of Approximation) plus its 90% confidence interval (CI), and the SRMR (Standardized Root Mean Square Residual). Since the χ^2^ is very sensitive to the sample size [37], *χ*^2^/*df* was used, with values less than 5 being considered acceptable [38]. Incremental indices (CFI and IFI) showed a good fit with values equal to or greater than 0.90 [39], while the error indices (RMSEA and SRMR) were considered acceptable with values equal to or less than 0.080 [40,41].

## 5. Study 2 Results

### 5.1. Descriptive Statistics, Reliability Analysis and Bivariate Correlations

The descriptive statistics, bivariate correlations and reliability analysis by Cronbach’s α can be seen in Table 4. The internal consistency analysis revealed Cronbach α values greater than 0.80 for each of the variables. Regarding the correlation analysis, it showed how the support for autonomy on the part of the teacher, friends and parents correlated negatively with psychological control by the teacher, friends and parents, and positively with respect to motivation towards physical activity, intention, attitude, behavioural control and subjective norms. In contrast, psychological control on the part of the teacher, friends and parents correlated negatively with regard to motivation towards physical activity, intention, attitude, behavioural control and subjective norms. Finally, the variables for motivation towards physical activity, intent, attitude, behavioural control and subjective norms correlated positively with each other.

### 5.2. Analysis of the Structural Equations Model

To test the SEM and analyse the relationships existing between the variables belonging to the proposed model. Previously, the number of latent variables present in those factors that were not composed of subfactors was reduced, this is particularly appropriate when the sample size is not too large compared to the number of model variables [47]. More specifically, the autonomy support (parents, teacher and friends), it was necessary to divide the 12 items on the scale into two indicators, as were the cases with the seven items pertaining to psychological control (parents, teacher and friends), the five items of perceived behavioural control, the four items of intention and the seven items of attitude. This procedure was followed to be able to identify the model, precisely as suggested by McDonald and Ho [48]. 

The hypothesized predictive relationship model (Figure 4) shows that the fit indices were appropriate: *χ*^2^ (223, N = 653) = 456.12, *χ*^2^/*df*= 2.05, *p* < 0.001, IFI = 0.97, CFI = 0.97, RMSEA = 0.045. (IC 90% = 0.036–0.051), SRMR = 0.030. The results conform to the established parameters so we can accept the proposed model as being appropriate [41]. Similarly, the contribution of each factor to the prediction of other variables was examined through standardized regression weights.

## 6. Discussion

The objective of this study has two parts—on the one hand, to adapt and validate the SPSEC-T, SPSEC-P and SPSEC-F to the Spanish context and, on the other, to analyse how the context of social support perceived by adolescents influences their own motivation and leads to the behaviour of physical activity practice.

The results demonstrate that the three instruments show evidence of validity and reliability, allowing us to measure the social support received by the person in relation to PA practice. In addition, the three instruments were invariant with respect to gender, allowing us to make comparisons between boys and girls. These instruments help us to delve into and understand the influence of social context on motivation and the adoption of certain behaviours linked to the practice of physical activity.

This study shows how the social context (the teacher, friends and parents) exerts a significant influence on adolescent motivation towards PA practice, except for autonomy support (teacher), which was not significant but in Pearson’s correlation analysis. The model found that support for autonomy exerts a positive influence on the motivation towards PA, while psychological control exerted a negative influence on the motivation towards PA. These results are similar to several previous studies conducted both nationally and internationally where the teacher’s influence on student motivation towards PA practice was analysed, showing that when teachers encouraged personal growth, the development of self-esteem and a sense of personal worth during their PA classes, the students had greater motivation towards the normal practice of PA [49,50,51,52]. Conversely, there are hardly any studies that analysed the influence of parents and friends on such motivation, let alone studies that considered both parents, friends and the teacher. Despite this, there are a number of studies in contexts other than PA in which they analysed the influence of friends and parents in adopting certain behaviours, taking into account the motivation of the adolescents. Some of these studies have focused on various contexts such as education and healthy habits [53,54,55,56], showing that close family and friendships are the nuclei in which the first experiences of socialization, protection and security originate, and are the origin of the adolescent’s future behaviours based on previous learning [57]. Therefore, it is understood that a social context favouring a controlling style to be articulated in most PA activities, blaming for not practising, reacting harshly or coercing to force physical exercise to be done, would result in a number of negative consequences regarding the adolescent’s interest and future PA practice. In contrast, a supporting style where autonomy is promoted, facilitating and promoting physical activity, providing advice and, at the same time, maintaining a responsive attitude, would positively encourage PA practice by the adolescent. In any case, the effects of both variables are antagonistic: while the perceived support for autonomy on the part of parents, teachers and friends favours motivation towards PA practice, the control of these same external agents reduces it.

On the other hand, the results showed how motivation towards the practice of physical activity has been positively related to attitude, subjective norms, behavioural control and intention, with the latter, in turn, being positively related to the weekly physical activity rate. These results can be discussed in relation to previous studies showing that a person’s high internal motivation towards the practice of activity is positively related to attitude, the intention to be physically active, and adopting a predisposition towards practice resulting from a positive mental representation towards such practice. A study conducted by Belando [58] on an adolescent population showed how motivation towards the practice of physical activity had a positive influence on attitude, behavioural control and subjective norms, favouring the intention to be physically active. Similarly, a study conducted on a population of adolescents by Hagger, Chatzisarantis and Biddle [59], analysing how intrinsic motivation, introjected regulation and external regulation were related to attitude, behavioural control and subjective norms showed that only intrinsic motivation was significant with regard to the previous three variables, and that it was also positive. In turn, attitude, behavioural control and subjective norms showed a positive relationship with respect to the intention to practice PA. Likewise, a study carried out on an adolescent population by [60] showed, via a SEM, that attitude, behavioural control and subjective norms positively predicted intention towards physical activity, and that this positively predicted the rate of physical activity performed by the adolescent.

However, as regards the findings from the model, it is necessary to emphasize that this is a correlational study so no cause–effect relationships can be extrapolated. Moreover, the study has sought to expose possibilities rather than causality so we can explain the relationships between the variables in both studies. The model seems to show good robustness and the ability to generalize about different cultures or ages; this helps us to better understand the role of the social context in motivation and the adoption of active behaviour regarding the practice of physical activity. However, future studies should analyse not only the cognitive and behavioural components but also the emotional component, given the importance of assessing adolescent emotional states in the practice of physical activity. In this way, we can better understand how to increase the likelihood of these people participating in physical activities outside of school and improve their quality of life by reducing the likelihood of unfavourable emotional experiences in this context.

## 7. Conclusions

In short, the results of this study show that the three scales referring to the social context (teachers, parents and friends) demonstrate acceptable fit indices. In addition, the SEM results fitted well with the data, with good representativeness existing between and of the factors. On the other hand, the results support the postulates established by the TPB, in which, at the beginning, Ajzen and Madden [61] developed the idea that the three components would affect behaviour through the effect mediated by intentions; in this regard, our results support some of the studies carried out in this area [33,34,58,60]. Moreover, based on the postulates of the SDT, the social context (parents, friends and teachers) is relevant in order for adolescents to develop an autonomous motivation towards the practice of physical activity. In addition, adolescents who have high autonomous motivation will show a predisposition towards manifesting adaptive behaviours since it is related to personal values, attitudes and objectives. Hence, adolescents will have a greater predisposition towards volitional behaviour, in this case, towards the practice of PA because they have the drive to achieve the objectives set for this behaviour.

## Figures and Tables

**Table 1:** Descriptive statistics, internal consistency analysis and bivariate correlations.

Factors	M	SD	Range	α	1	2	3	4	5	6
1. Autonomy support (Parents)	4.88	1.23	1–7	0.83		−0.45 **	0.27 **	−0.23 **	0.42 **	−0.27 **
2. Psychological Control (Parents)	2.31	1.07	1–7	0.81			−0.22 *	0.37 **	−0.37 **	−0.25 *
3. Autonomy support (Friends)	5.34	1.29	1–7	0.88				−0.31 **	0.32 **	−0.21 **
4. Psychological Control (Friends)	1.94	1.12	1–7	0.87					−0.34 **	0.42 **
5. Autonomy support (Teacher)	4.45	1.32	1–7	0.90						−0.28 **
6. Psychological Control (Teacher)	2.13	1.03	1–7	0.84						

Note: ** *p* < 0.01; * *p* < 0.001; M = Mean; SD = Standard Deviation.

**Table 2:** Gender Invariance Analysis.

**Perceived Support in the Contexts of Exercise on the Part of the Teacher**									
Models	χ^2^	*df*	χ^2^/*df*	Δχ^2^	Δ*df*	CFI	IFI	RMSEA (IC 90%)	SRMR
Model 1	753.12	302	2.49	-	-	0.93	93	0.059 (0.054–0.065)	0.041
Model 2	769.93	319	2.41	16.82	17	0.93	0.93	0.058 (0.052–0.063)	0.042
Model 3	826.83	341	2.42	73.71 **	36	0.93	0.93	0.058 (0.053–0.063)	0.044
Model 4	861.97	360	2.39	108.86 ***	58	0.93	0.93	0.058 (0.053–0.063)	0.049
**Perceived Support in the Contexts of Exercise on the part of the Parents**									
Models	χ^2^	*df*	χ^2^/*df*	Δχ^2^	Δ*df*	CFI	IFI	RMSEA (IC 90%)	SRMR
Model 1	864.46	302	2.86	-	-	0.92	0.92	0.066 (0.061–0.071)	0.044
Model 2	876.06	319	2.75	11.60	17	0.92	0.92	0.064 (0.059–0.069)	0.043
Model 3	893.57	341	2.62	29.11	36	0.92	0.92	0.062 (0.057–0.067)	0.046
Model 4	960.02	360	2.67	95.56 **	58	0.91	0.91	0.063 (0.058–0.067)	0.052
**Perceived Support in the Contexts of Exercise on the Part of Friends**									
Models	χ^2^	*df*	χ^2^/*df*	Δχ^2^	Δ*df*	CFI	IFI	RMSEA (IC 90%)	SRMR
Model 1	844.55	302	2.80	-	-	0.94	0.94	0.065 (0.060–0.070)	0.034
Model 2	854.66	319	2.68	4.42	17	0.94	0.94	0.063 (0.05–80.068)	0.035
Model 3	950.68	341	2.79	106.13 ***	39	0.94	0.94	0.065 (0.059–0.069)	0.043
Model 4	1118.31	360	3.11	173.76 ***	58	0.92	0.92	0.070 (0.066–0.075)	0.047

** *p* < 0.01; *** *p* < 0.001. Please note: Model 1 = the unrestricted model; Model 2 = the measurement weight invariance model; Model 3 = the invariant structural covariance model; Model 4 = the residual invariance measurement model. Note: CFI (Comparative Fit Index), IFI (Incremental Fit Index), RMSEA (Root Mean Square Error of Approximation), and SRMR (Standardized Root Mean Square Residual).

**Table 3:** Age Invariance Analysis.

**Perceived Support in the Contexts of Exercise on the Part of the Teacher**									
Models	*χ* ^2^	*df*	*χ*^2^/*df*	Δχ^2^	Δ*df*	CFI	IFI	RMSEA (IC 90%)	SRMR
Model 1	772.35	302	2.55	-	-	0.94	94	0.060 (0.055–0.068)	0.040
Model 2	781.21	319	2.44	18.35	17	0.94	0.94	0.059 (0.055–0.064)	0.043
Model 3	854.96	341	2.50	68.37 **	36	0.94	0.94	0.059 (0.051–0.064)	0.048
Model 4	872.11	360	2.42	98.56 **	58	0.94	0.94	0.059 (0.051–0.064)	0.053
**Perceived Support in the Contexts of Exercise on the Part of the Parents**									
Models	*χ* ^2^	*df*	χ^2^/*df*	Δχ^2^	Δ*df*	CFI	IFI	RMSEA (IC 90%)	SRMR
Model 1	802.77	302	2.65	-	-	0.93	0.93	0.065 (0.058–0.068)	0.043
Model 2	828.16	319	2.59	15.71	17	0.93	0.93	0.064 (0.059–0.067)	0.046
Model 3	855.39	341	2.51	24.42 **	36	0.93	0.93	0.064 (0.059–0.067)	0.049
Model 4	902.85	360	2.51	92.56***	58	0.93	0.93	0.061 (0.056–0.063)	0.051
**Perceived Support in the Contexts of Exercise on the Part of Friends**									
Models	*χ* ^2^	*df*	*χ*^2^/*df*	Δχ^2^	Δ*df*	CFI	IFI	RMSEA (IC 90%)	SRMR
Model 1	830.45	302	2.75	-	-	0.94	0.94	0.065 (0.061–0.069)	0.041
Model 2	849.32	319	2.66	9.74	17	0.94	0.94	0.064 (.059–0.067)	0.045
Model 3	921.87	341	2.70	89.32 *	39	0.94	0.94	0.062 (.060–0.065)	0.046
Model 4	992.19	360	2.76	113.78 **	58	0.93	0.93	0.062 (.060–0.066)	0.047

* *p* < 0.05; ** *p* < 0.01; *** *p* < 0.001. Please note: Model 1 = the unrestricted model; Model 2 = the measurement weight invariance model; Model 3 = the invariant structural covariance model; Model 4 = the residual invariance measurement model.

**Table 4:** Descriptive statistics, internal consistency analysis and bivariate correlations.

Factors	*M*	*SD*	Range	α	1	2	3	4	5	6	7	8	9	10	11	12
1. Autonomy support (Parents)	5.29	1.34	1–7	0.93		−0.22 **	0.69 **	−0.15 **	0.39 **	−0.16 **	0.57 **	0.43 **	0.55 **	0.64 **	0.53 **	0.43 **
2. Psychological Control (Parents)	2.32	1.13	1–7	0.98			−0.13 *	0.04	−0.45 **	−0.14 *	−0.13 **	−0.13 *	−0.14 *	−0.12 **	−0.13 **	−0.10
3. Autonomy support (Friends)	5.12	1.24	1–7	0.96				−0.10 *	0.39 **	−0.13 *	0.48 **	0.39 **	0.43 **	0.55 **	0.52 **	0.38 **
4. Psychological Control (Friends)	2.18	1.05	1–7	0.98					−0.20 **	0.32 **	−0.46 *	−0.12 *	−0.12 *	−0.16 **	−0.14	−0.03
5. Autonomy support (Teacher)	4.78	1.40	1–7	0.94						−0.12 *	0.30 **	0.23 **	0.16 **	0.38 **	0.21 **	0.17 **
6. Psychological Control (Teacher)	2.02	1.17	1–7	0.83							−0.30 **	−0.10	0.12 *	−0.13 *	−0.19 **	−0.07 *
7. Motivation Physical Activity	2.90	1.65	1–7	-								0.41 **	0.48 **	0.47 **	0.54 **	0.36 **
8. Attitude	5.55	1.69	1–7	0.96									0.42 **	0.48 **	0.48 **	0.20 **
9. Behavioural Control	5.27	1.41	1–7	0.92										0.51 **	0.70 **	0.46 **
10. Subjective Norms	5.30	1.32	1–7	0.85											0.57 **	0.36 **
11. Intention	5.23	1.31	1–7	0.88												0.53 **
12. Physical Activity	3.84	1.21	1–7	-												

* *p* < 0.05; ** *p* < 0.01. Note: M = Mean; SD = Standard deviation; α = Cronbach’s α.

## Appendix A

La escala está precedida por el siguiente encabezamiento: “En relación a la práctica de actividad física, mi profesor de EF/amigos/padres…”

*- The scale is preceded by the following heading: “In relation to the practice of physical activity, my PE teacher/friends/parents…”*

**1.** Me facilita con distintas opciones cómo realizar el ejercicio físico o deportivo en mi tiempo libre.

*- It facilitates me with different options how to carry out the physical exercise or sport in my free time.*

**2.** Siempre está tratando de cambiarme.

*- He’s always trying to change me.*

**3.** Entiende por qué decido hacer ejercicio físico en mi tiempo libre

*- Understand why I decide to exercise in my free time*

**4.** Me hace sentir culpable cuando no logro cumplir sus expectativas

*- Makes me feel guilty when I fail to meet your expectation*

**5.** Confían en mi capacidad de hacer ejercicio físico o deportivo en mi tiempo libre

*- They trust my ability to do physical exercise or sports in my free time.*

**6.** Se muestra menos cercano, si no hago lo que pide

*- Shows less close, if I don’t do what you ask*

**7.** Me animan a practicar algún ejercicio físico o deportivo en mi tiempo libre

*- I am encouraged to practice some physical exercise or sport in my free time.*

**8.** Reacciona con dureza cuando lo decepciono

*- He reacts harshly when I disappoint him.*

**9.** Escucha mis comentarios sobre el ejercicio físico o deportivo que realizo en mi tiempo libre

*- Listen to my comments about the physical or sports exercise I do in my free time.*

**10.** Me hace sentir culpable cuando no está satisfecho con mi trabajo

*- Makes me feel guilty when I’m not satisfied with my work*

**11.** Me anima de forma positiva cuando hago ejercicio físico o deportivo en mi tiempo libre

*- It encourages me in a positive way when I do physical exercise or sports in my free time.*

**12.** Evita hablarme cuando lo he decepcionado

*- Avoid talking to me when I have disappointed you*

**13.** Soy capaz de dirigirme hablándole sobre el ejercicio físico o deportivo que hago en mi tiempo libre

*- I am able to talk to you about the physical exercise or sports that I do in my free time.*

**14.** Soy capaz de compartir mis experiencias de ejercicio físico o deportivo

*- I am able to share my experiences of physical exercise or sports*

**15.** A menudo me interrumpe

*- …often interrupts me*

**16.** Se asegura de entender por qué tengo que hacer ejercicio físico o deportivo en mi tiempo libre

*- Make sure you understand why I have to do physical exercise or sports in my free time.*

**17.** Contesta a mis preguntas sobre el ejercicio físico o deportivo que realizo en mi tiempo libre

*- Answer my questions about the physical exercise or sports that I do in my free time.*

**18.** Se preocupa por el ejercicio físico o deportivo que realizo en mi tiempo libre

*- Worry about the physical or sports exercise I do in my free time*

**19.** Confío en el consejo que me da sobre el ejercicio físico o deportivo que hago en mi tiempo libre

*- I trust the advice you give me about the physical exercise or sports I do in my free time.*

Note: The scale was validated in Spanish
